# Repeated episodes of spontaneous regression/progression of cervical adenocarcinoma after adjuvant chemoradiation therapy: a case report

**DOI:** 10.1186/s13256-015-0578-8

**Published:** 2015-05-19

**Authors:** Atsuto Katano, Ryousuke Takenaka, Kae Okuma, Hideomi Yamashita, Keiichi Nakagawa

**Affiliations:** Department of Radiation Oncology, University of Tokyo Hospital, 7-3-1, Hongo, Bunkyo-ku, Tokyo 113-8655 Japan

**Keywords:** Abscopal effect, Cervical cancer, Radiation therapy, Spontaneous regression

## Abstract

**Introduction:**

Spontaneous regression of cancer is thought to be a rare event. Here, we report an extremely rare case of repeated episodes of spontaneous regression and progression of recurrent cervical adenocarcinoma.

**Case presentation:**

We report here a case of a 56-year-old Japanese woman who was diagnosed with cervical adenocarcinoma. Her hilar and mediastinal lymph nodes were swollen 6 years after the initial diagnosis and subsequent treatment, and were found to be pathologically malignant by mediastinal biopsy. Then, without any treatment, the hilar and mediastinal lymph nodes spontaneously regressed with decreases in tumor size and serum tumor marker levels, as confirmed by a decrease in uptake of fluorodeoxyglucose during positron emission tomography-computed tomography. Subsequently, although there were repeated episodes of increase and decrease in her serum tumor marker levels and lymph node size, her activities of daily living were and are well preserved.

**Conclusions:**

While spontaneous regression of a malignant tumor is a rare event, our case is even rarer in that repeated episodes of spontaneous regression/progression of cervical adenocarcinoma occurred.

## Introduction

The widely adopted definition of spontaneous regression of cancer is “the partial or complete disappearance of a malignant tumor in the absence of treatment or in the presence of therapy considered inadequate to exert a significant influence on the disease” as proposed by Everson and Cole in 1966 [1]. Spontaneous regression of cancer is thought to be a rare event with an incidence of one in 60,000 to 100,000 [2], and more than half of these events occur in malignant melanoma, renal cell carcinoma, hematopoietic cancer, and neuroblastoma [3]. Here, we report an extremely rare case of repeated episodes of spontaneous regression and progression of recurrent cervical adenocarcinoma.

## Case presentation

**Figure 1:**
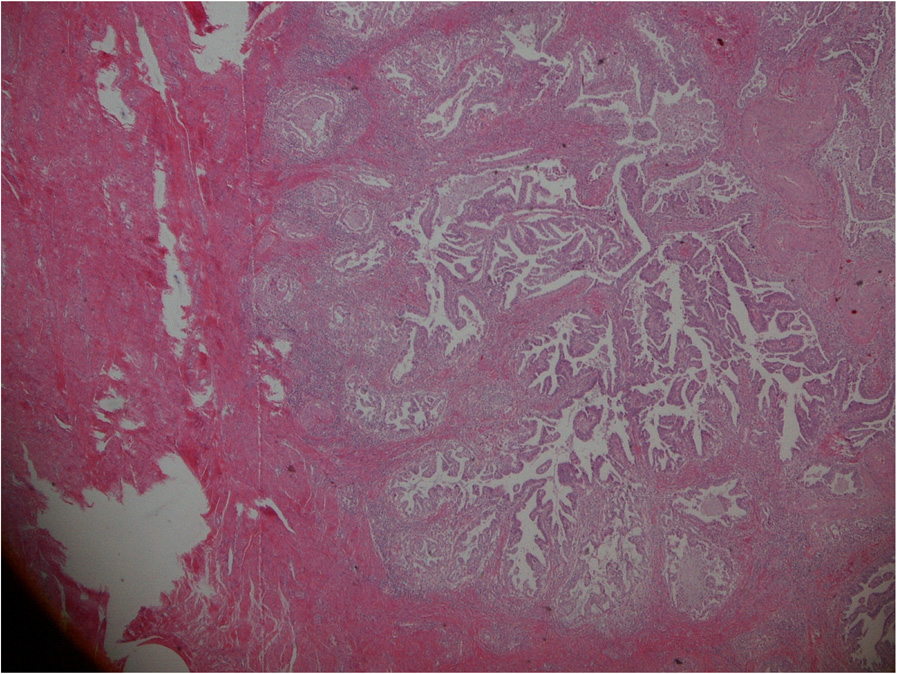
Histopathological analysis of the biopsy sample taken during the patient’s first visit. It revealed an adenocarcinoma involving the uterine cervix. Small cells are arranged in a dense papillary or tubular growth pattern. A portion of tumor cells remain regular glands. Hematoxylin and eosin stain, low power.

A 56-year-old Japanese woman was referred to the gynecology department at our hospital with complaints of vaginal bleeding in August 1997. She was found to have a cervical mass, which was biopsied and diagnosed as an adenocarcinoma (Figure 1). A pelvic examination revealed a 1 to 2cm cervical mass invading her vagina on the posterior lip of her cervix. On rectovaginal examination, the parametria and posterior fornix seemed to be free of disease. Her serum tumor markers were elevated: carcinoembryonic antigen (CEA) was 10.5ng/mL (<5ng/mL), carbohydrate antigen 19–9 (CA19-9) was 151U/mL (<37U/mL), and carbohydrate antigen 125 (CA-125) was 343U/mL (<30U/mL). T2-weighted pelvic magnetic resonance imaging showed a cervical lesion of 1×1.5cm without parametrial invasion, lymphadenopathy or metastasis, compatible with the International Federation of Gynecology and Obstetrics stage IIa. Total abdominal hysterectomy with bilateral salpingo-oophorectomy and pelvic lymphadenectomy were performed in September 1997. The final pathology demonstrated a 1.5×1.5×1.0cm highly differentiated papillary adenocarcinoma with myometrial invasion confined to less than one-half of her uterine wall, and no evidence of lymph vascular space invasion. The surgical margins were free of cancer. A total of 36 lymph nodes were dissected; one of the lymph nodes, located in the left iliac lymph node, was positive for disease, compatible with pathological stage T2aN1M0. Based on pathologic risk factors, we recommended her for adjuvant chemoradiation therapy. She was administered two 21-day courses of chemotherapy comprising a combination of cisplatin (110mg/body) on day 1, mitomycin C (11mg/body) on day 1, and etoposide (150mg/body) on days 1, 3, and 5, followed by pelvic radiation therapy; the clinical target volume included the uterus, cervix, vagina, as well as the presacral, obturator, and external, internal, and common iliac lymph nodal groups: 10MV X-ray parallel opposed pair two-field box and 50Gy in 2Gy daily fractions were used.

The serum tumor marker levels decreased significantly after the initial treatment, and she showed no signs of recurrence. From June 1998, she needed short hospitalization repeatedly because she had symptoms of ileus possibly induced by surgical operation or radiotherapy, and transverse colectomy was performed at our hospital in August 1999. She had an uneventful postoperative course and has not been hospitalized for further abdominal problems since then.

**Figure 2:**
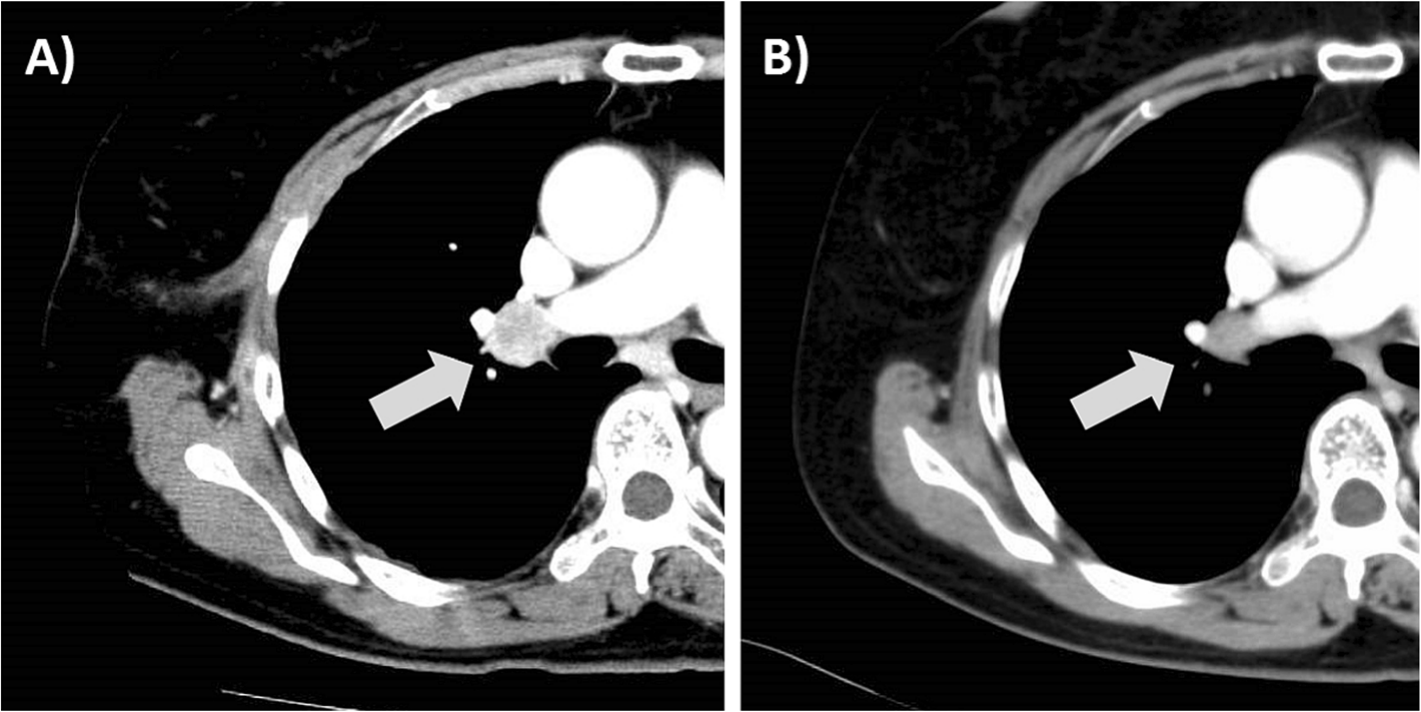
Hilar lymph node metastases appeared 6 years after initial treatment of the primary tumor. **A)** A contrast-enhanced computed tomography image of the patient’s chest performed in July 2003. The arrow indicates a contrast-enhancing enlarged lymph node. Serum tumor markers are elevated as follows: carcinoembryonic antigen was 43.8ng/mL, carbohydrate antigen 19–9 was 151U/mL, and carbohydrate antigen 125 was 343U/mL in June 2003. **B)** The lymph node (arrow) decreased to 65% of its peak volume in June 2005, 2 years after the recurrence. Serum tumor markers were decreased as follows: carcinoembryonic antigen was 27.1ng/mL, carbohydrate antigen 19–9 was 27U/mL, and carbohydrate antigen 125 was 188U/mL.

**Figure 3:**
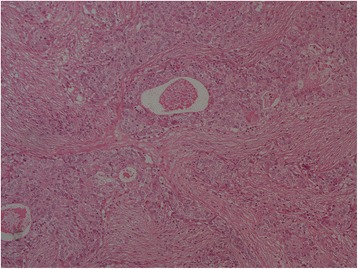
Histopathological analysis of the mediastinal lymphadenopathy in July 2003. The sample thought to be a recurrent lesion revealed a moderately to poorly differentiated adenocarcinoma. The tumor cells with round nuclei, eosinophilic cytoplasm, and distinct cell borders are arranged in sheets and show alveolar or solid growth pattern with some glandular cavity. A large number of tumor cells have mucinous cytoplasm. The histologic examination of the primary lesion, which is diagnosed as a “well-differentiated” adenocarcinoma, is inconsistent with the recurrent lesion. However, inconsistent histological analysis between metastatic and primary tumors often occurs. The primary lesion also contains a sheet-like structure, so there is some consistency in diagnosis of this lesion as a recurrence. Hematoxylin and eosin stain, low power.

Her serum tumor markers gradually elevated from May 2002: CA-125 was 93U/mL, CEA was 11.7ng/mL, and CA19-9 was 15U/mL in May 2002; CEA was 21.3ng/mL, CA19-9 was 23U/mL, and CA-125 was 114U/mL in November 2002; and CA-125 was 344U/mL, CEA was 47.5ng/mL, and CA19-9 was 139U/mL in April 2003. In May 2003, a computed tomography (CT) scan revealed bilateral hilar and mediastinal lymphadenopathy (Figure 2A). Biopsy of the mediastinal lymph nodes was done in July 2003. She was diagnosed with adenocarcinoma, consistent with recurrence (Figure 3). We recommended salvage chemotherapy to our patient, but she was not compliant with the recommendations and the treatment was never delivered. Without treatment, the serum tumor marker levels gradually decreased: CEA was 52.8ng/mL, CA19-9 was 124U/mL, and CA-125 was 231U/mL in January 2004; and CEA was 32.4ng/mL, CA19-9 was 33U/mL, and CA-125 was 180U/mL in January 2005. A CT scan also demonstrated a reduction in size of the recurrent lesions (mediastinal and bilateral hilar lymph nodes) in June 2005 (Figure 2B).

In October 2006, her serum tumor markers were again highly elevated: CA-125 was 134U/mL, CEA was 39.3ng/mL, and CA19-9 was 138U/mL. Further, positron emission tomography-CT (PET-CT) imaging revealed a new lesion located in her right supraclavicular lymph node (maximum standardized uptake value, SUV max, 6.0) in addition to the existing lesions in her mediastinal (SUV max 6.0) and bilateral hilar (SUV max 14.9) lymph nodes. Although she remained untreated, PET-CT imaging in October 2008 revealed that her right supraclavicular lymph node had no significant uptake and the other lesions had decreased uptake of fluorodeoxyglucose (^18^F-FDG; mediastinal lymph node, SUV max 9.2; right hilar lymph node, SUV max 5.0; left hilar lymph node, SUV max 3.0). A new swollen lymph node with a size of 8.6cm^3^ on CT scan appeared in her right axillary lymph node in September 2012, but it was reduced to 3.4cm^3^ in February 2014. A 15mm-diameter nodule, which was observed in the S2 segment of her right upper lobe on CT scan in February 2014 and formed a cavity, reduced in volume by July 2014. She is currently in good condition without any cancer treatment from the time of recurrence and has survived for about 18 years after the initial diagnosis. Her performance status is still excellent (Karnofsky performance status score, 100%).

**Figure 4:**
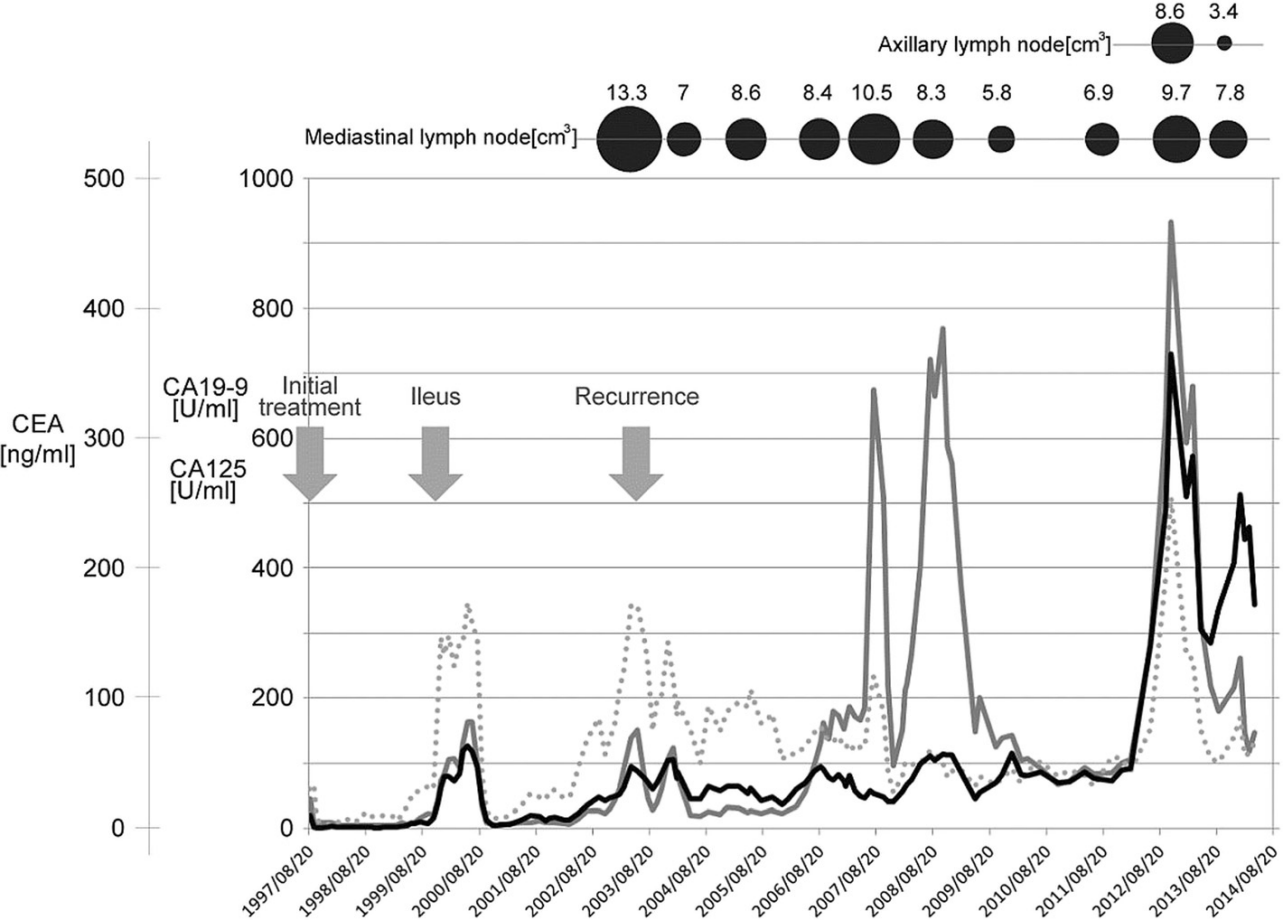
Changes in serum tumor markers and volume of the axillary and mediastinal lymph nodes. The changes in serum tumor markers (carbohydrate antigen 19–9, carbohydrate antigen 125, and carcinoembryonic antigen) and volume of the axillary and mediastinal lymph nodes from initial presentation until present. The black solid line indicates the serum level of carcinoembryonic antigen, the gray solid line indicates the serum level of carbohydrate antigen 19–9, and the gray dotted line indicates the serum level of carbohydrate antigen 125. Abbreviations: CA, carbohydrate antigen; CEA, carcinoembryonic antigen.

A follow-up examination included a physical examination, assessment of tumor markers, and CT scan. Figure 4 shows the changes in serum tumor markers (CA19-9, CA-125, and CEA) and volume of the axillary and mediastinal lymph nodes.

## Discussion

Our case met Everson and Cole’s definition since she experienced partial regression of the lesion that was diagnosed as malignant without any medical treatment. There are several presumed mechanisms of spontaneous regression: (1) immunological mechanism, (2) hormone-induced stimulation, (3) impact of infectious diseases, (4) traumatic effect of surgical operation, (5) nutritional/circulatory deficiency of tumor, (6) abrasion of superficial tumors, (7) elimination of carcinogen or tumor growth factor, (8) psychotherapy or alteration of mental state, and (9) complementary and alternative medicine [4-6]. Immunological mechanism is thought to be the most important factor, and the study of anti-tumor immune responses has received enhanced interest in recent years.

Programmed cell death 1 (PD-1), which is expressed on the surface of T lymphocytes, and its ligand (programmed death-ligand 1; PD-L1) negatively regulate the immune response [7]. Many tumor cells express PD-L1 and protect themselves from the body’s immune system. Immunotherapy via inhibition of the interaction between PD-1 and PD-L1 by anti-PD-1 antibody is effective in advanced melanoma and kidney cancer [8]. We suspect that the spontaneous regression process in our case was a result of the ongoing competition between the immune system and tumor progression; when the anti-tumor response of the immune system became predominant, the cancer regressed, and when the immune system was weakened, the cancer progressed. This balance of the opposing forces probably led to the repeated episodes of regression and progression rather than complete remission. The exact situation in our patient and the mechanisms that govern these opposing forces are unclear, but the PD-1/PD-L1 anti-tumor immune system may be involved. We are considering performing immunostaining for PD-L1 on future biopsy samples from the patient to shed more light on this mechanism.

In our case as there were a limited number of relapsed sites, called oligometastases or oligo-recurrence [9], the immunological response may have been caused by an abscopal effect, a phenomenon in which local radiotherapy is associated with the regression of a metastatic lesion that is located at a distance from the irradiated site. A recent report showed that irradiation of the mediastinum resulted in regression of an untreated lung tumor [10]. In a patient with advanced uterine cervical carcinoma, para-aortic lymph node metastasis showed an abscopal effect of radiation therapy [11]. While radiotherapy has been predominantly focused on direct damage to malignant cells, there is increasing evidence that it can also lead to significant alterations in the tumor microenvironment, particularly with respect to effects on immune cells and infiltrating tumors [12]. A recent study indicates that the abscopal effect might have the potential to affect disappearance of micrometastases [13]. It is not typical that the abscopal effect is so long-lasting considering the long interval between the time of irradiation (1997) and spontaneous regression (2004). However, it is highly possible that the abscopal effect activated an anti-tumor response of the immune system in our patient that resulted in the repeated episodes of tumor regression and progression.

## Conclusions

Spontaneous regression has intrigued clinicians for many years; however, little is known about why it occurs. Our patient demonstrated an intriguing clinical course of repeated episodes of spontaneous regression/progression of pathologically confirmed malignant lesions. Despite the unknown underlying causes of spontaneous regression, the potential contribution of such knowledge is thought to be essential to treatment or prevention of cancer.

## Consent

Written informed consent was obtained from the patient for publication of this case report and accompanying images. A copy of the written consent is available for review by the Editor-in-Chief of this journal.

## Abbreviations

^18^F-FDG: Fluorodeoxyglucose
CA-125: Carbohydrate antigen 125
CA19-9: Carbohydrate antigen 19–9
CEA: Carcinoembryonic antigen
CT: Computed tomography
PD-1: Programmed cell death 1
PD-L1: Programmed death-ligand 1
PET-CT: Positron emission tomography-computed tomography
SUV max: Maximum standardized uptake value
